# Flickering flash signals and mate recognition in the Asian firefly, *Aquatica lateralis*

**DOI:** 10.1038/s41598-023-29552-6

**Published:** 2023-02-10

**Authors:** Hideo Takatsu, Mihoko Minami, Yuichi Oba

**Affiliations:** 1 5-58 Takayokosuka-machi, Tokai, Aichi 477-0037 Japan; 2 Department Environmental Biology, Chubu University, 1200 Matsumoto-cho, Kasugai, Aichi 487-8501 Japan; 3 Department of Mathematics, Keio University, 3-14-1 Hiyoshi, Kohoku-ku, Yokohama, Kanagawa 223-8522 Japan

**Keywords:** Behavioural ecology, Entomology

## Abstract

Nocturnal fireflies sometimes use intricate bioluminescent signal systems for sexual communication. In this study, we examined flash signals and mate recognition in the Asian firefly, *Aquatica lateralis*, under natural field conditions. We found that the flash pattern of females changes after copulation, from simple short flashes to flashes with longer duration and flickering. To understand the functions of flickering, we video-recorded and analyzed the flashes of sedentary males, receptive females, and mated females. The results showed that the flashes of these three adult phases can be discriminated from each other by two parameters, flash duration and flicker intensity, with little overlap. Male attraction experiments using an artificial LED device termed ‘e-firefly’ confirmed that flying and sedentary males are attracted to flashes with shorter durations and lower flicker intensities. The range of attraction success was much wider for flying males and narrower for sedentary males, and the latter was close to the range of receptive female’s flashes. These findings suggest that in addition to flash duration, flicker intensity is a flash signal parameter of mate recognition in *A. lateralis* males.

Fireflies, the species in the family Lampyridae, are distributed in most temperate and tropical regions of the world. There are over 2000 currently recognized species, and all are considered to be bioluminescent at least in the larval stages, while only a portion of the nocturnal species are bioluminescent in the adult stage^1^. Based on molecular phylogenetic analyses, it is estimated that the original function of luminescence in Lampyridae was warning display to exhibit toxicity or distastefulness at the larval stages, and the function of mating communication in the adult stage was secondarily acquired several times during evolution^2,3^.

To understand the flash signal system for mating in nocturnal firefly species, two critical studies were conducted in the early twentieth century; McDermott^4^ and Mast^5^ performed the field observations and laboratory experiments of the North American common firefly *Photinus pyralis* (subfamily Lampyrinae), showing that the primary role of male flashes is to serve as a stimulator of female flash response (male advertisement). Later, Buck identified using an artificial light device that the timing of the response delay to the male’s signal by the female is an essential parameter of mate recognition by the male^6^. Firefly signal systems for mating have been further studied using other ‘dialog’ firefly species in North America and Europe, and various other signal parameters, such as the flash duration (FD) and pulse interval of male flashes, have been identified^7–9^.

‘Female advertising’ has been reported in some Asian firefly species, in which the female is the photic advertiser and the male is the receiver^10^. We previously investigated the signal parameters of female calls using the Asian female-advertising firefly, *Luciola parvula* (subfamily Luciolinae)^10^. Based on the field observation of male/female signals and male attraction tests using an electronic LED device, we elucidated that a signal parameter for mate recognition is FD; the female flashes spontaneously, and the male discriminates the flashes of males and females based on FD. This was the first example used to determine the signal parameter in female-advertising fireflies^10^.

The Asian firefly, *Aquatica lateralis* (subfamily Luciolinae), is distributed in Siberia, Northeast China, the Kuril Islands, Korea, and mainland Japan (Hokkaido, Honshu, Shikoku and Kyushu)^11^. The whole-genome assembly for this species has been published, and laboratory culture techniques have also been established^12^; thus, *A. lateralis* is the most-established model firefly for postgenomic research^13^. The larvae are aquatic living in still or slow streams near rice paddies or wetlands and feed mainly on freshwater snails. In adults, both males and females are fully winged and can fly. They are nocturnal and use yellowish green bioluminescence for sexual communication^14^. During the active period at dusk, sexually receptive females (hereafter called ‘receptive females’), are sedentary, staying on the ground or vegetation, and emit short flashes at approximately 1 s intervals^15^. The females’ flashes are spontaneous; thus, *A. lateralis* is also considered to be a female-advertising firefly^10^. The males fly emitting flashes at 0.5–1.0 s intervals to search for sedentary females’ flashes and then land near the flashing female. Some males do not fly and search for females on the ground or vegetation during the active period. We hereafter call those landed males and sedentary males ‘sedentary males.’ Each flash by the sedentary male shows characteristic ‘flickering’ or ‘twinkling’ in the millisecond range^14^.

The apparent ‘flickering’ flash has also been recognized in some species in all major subfamilies in Lampyridae—Luciolinae, Lampyrinae, and Photurinae^14–21^. In *A. lateralis*, Ohba hypothesized that flickering by sedentary males is an inducer of female calling behavior^14^. However, the function of flickering as a flashing signal has not been examined empirically in any firefly species. To understand the function of the flickering component of the firefly’s flash, in the present study, we first analyzed the flash patterns of male and female *A. lateralis* during mating behavior. Then, we conducted male attraction experiments using a programmed LED light device named e-firefly. Based on the data analysis, we concluded that the flicker intensity, in addition to the flash duration, is a flash signal parameter of mate recognition in *A. lateralis* males.

## Materials and methods

### Flash recording

All field recording and experiments were performed at the paddy field in the Northern Chita Peninsula, Aichi Prefecture, central Japan, in June and July between 2003 and 2016. The ambient temperature at the firefly’s active period was measured using a thermometer. The flashes were recorded with a digital video camera (NV-GS-400, Panasonic, Japan) mounted on a tripod at a height of 30–50 cm from ground and a distance of 1.0–1.5 m away from the specimen. Isolated specimens were selected for recording to exclude the background light from other nontarget specimens. When another specimen appeared near the target specimen, the video recording was cancelled. When a female copulated during video recording in the field, her flashes until 1 min before copulation were regarded as those of a ‘receptive female’. To record the flashes of a ‘mated female’, the female specimens already mated were prepared in aquariums (because virgin and mated females cannot be distinguished in the field): the eggs were obtained from wild female specimens collected one year before at the same field and reared to adults; immediately after emergence the virgin female was confined in a small container with two cultured males for two nights to facilitate copulation. As the parents of the reared specimens were collected from the observation field (same genetic background), the rearing temperature was almost the same as that of the natural field, the emergence period of the cultured specimens overlapped with that of the natural population, the adult body sizes of the reared and natural specimens were indistinguishable, and the flash pattern of the cultured mated females was indistinguishable from that of the wild (potentially) mated females. Thus, we believe that there was no influence of different rearing environments, i.e., the flash behavior of the cultured mated female specimens is expected to be substantially the same as that of wild mated female specimens. To distinguish them from wild (potentially) mated females, the elytra of cultured mated females were marked with colored ink before placing them in the field, and after three days, the flashes of ink-marked specimens were recorded. Of note, we never observed male attraction and copulation in any of the mated females used for field observation; thus, the mated females were unreceptive.

### Waveform analysis

Sequential still images were captured from video files at 30 frames per second using VirtualDub (GPL), and then the light intensities in the images were qualified (8-bit linear gray scaling from black to white at 0–255) using ImageJ software. In this study, we defined ‘flash’ as a luminescent waveform from baseline to baseline and ‘flickering’ as fluctuation above baseline in a single flash. The waveforms containing a saturated signal (255, white) were omitted. The waveforms of the maximum signal value lower than 50 were also omitted because of the difficulty in separating signal and noise. Approximately 10–90 waveforms per individual were analyzed; thus, the effect of the occasional interruption of the flash recording by the specimen’s movement and/or vegetation swinging between the specimen and the video camera is statistically ignorable. FD is defined as the time interval between the beginning and the end of a flash (Fig. S1). Flicker intensity (FI) was defined as

$${\text{FI}} = \left\{ {\begin{array}{*{20}l} {\mathop {\max }\limits_{1 \le i \le n} \left( {\frac{{{\text{min}}\left( {p_{i} ,p_{i + 1} } \right) - t_{i} }}{{\min \left( {p_{i} , p_{i + 1} } \right) + t_{i} }}} \right)} \hfill & {{\text{if}} \, n \ge 1} \hfill \\ 0 \hfill & {{\text{if}} \, n = 0} \hfill \\ \end{array} } \right.$$

where p, t, and *n* denote the peak and the trough (local extrema) in the waveform of a flash and the number of toughs in the flash, respectively (Fig. S1). In total, we measured the FD and FI values of 347, 94, and 355 waveforms from 13 sedentary males, 7 receptive females, and 8 mated females, respectively. We did not consider the flash brightness as a factor because the measured value of the light intensity depends largely on the distance between the light source and the detector; thus, the actual brightness of the lantern cannot be practically measured in the field.

### e-Firefly

For male attraction experiments, we built an electronic LED device, the e-firefly, to generate patterned flashes with various FDs and FIs using a chip LED (green type, λmax = 568 nm, Everlight Electronics, Taiwan; Figs. S2 and S3) with a microcontroller PIC16F628A (Microchip Technology, USA) (see Figs. S4-S5). An example of the program for the microcontroller is shown in Supplementary Data S1. The brightness was constant in all programs. Flickering frequency ranged between 5–12 Hz, which corresponds to that of sedentary male flashes (approximately 10 Hz)^15^. To prevent direct access of the attracted specimen to the light source, the chip LED was covered by a steel net painted green (see Fig. S2). For flying male attraction experiments, when the male landed within a 100-mm distance from the e-firefly, we judged the attraction to be a success; otherwise, it was a failure. For sedentary male attraction experiments, the e-firefly was placed 200–300 mm away from the sedentary male. When the approaching male touched the steel net covering the e-firefly, to warrant a positive approach, we measured the time the male remained on the net. If the male did not move away from the net for more than 2 min, we judged the attraction to be a success (strict criterion for judgment); otherwise, it was a failure.

### Spectral measurement

The luminescence spectra of e-firefly and *A. lateralis* were measured using a Flame-S spectrophotometer (Ocean Insight, USA). The living *A. lateralis* specimens were anesthetized on ice and frozen at − 20 °C until use. The lantern started luminescence by thawing at room temperature, and the spectrum was measured during luminescence (within 5 min).

### Statistical analysis

First, we considered a discriminant analysis using a logistic regression model that discriminates between receptive females and others in the observational data. We fitted several models with combinations of FD and FI, quadratic terms of FD and FI (FD^2^, FI^2^), interaction of FD and FI (FD $\times$ FI), and temperature (T) as explanatory variables. Based on Akaike’s information criteria (AIC) values and model simplicity, we chose the logistic regression model with FD, FI, FD^2^ and T as explanatory variables. Let $p$(${\varvec{x}}$) denote the conditional probability that a flash is from a receptive female given ${\varvec{x}}=\left(\mathrm{FD},\mathrm{ FI},\mathrm{ T}\right)$ and $\widehat{p}$(${\varvec{x}}$) denote its estimate. The coefficients of the logistic regression model are estimated as follows.

**[Model for the observational data with temperature (T)]**

$$\begin{gathered} {\text{log}}\frac{{\hat{p}}}{{1 - \hat{p}}} = \begin{array}{*{20}l} { - 32.26 + 69.69 \times FD - 43.47 \times FI - 76.63 \times FD^{2} + 0.87 \times T} \hfill \\ {~\quad \left( {6.50} \right)\quad \quad \left( {15.37} \right)\quad \quad \quad \left( {8.56} \right)\quad \quad \quad \quad \left( {17.44} \right)\quad \quad \quad \left( {0.19} \right)~~} \hfill \\ \end{array} \hfill \\ \quad {\text{AIC: 84}}{\text{.75}} \hfill \\ \end{gathered}$$

**[Model for the observational data without temperature (T)]**

$$\begin{gathered} {\text{log}}\frac{{\hat{p}}}{{1 - \hat{p}}} = \begin{array}{*{20}l} { - 7.69~ + 47.57 \times FD~ - 38.29 \times FI~ - 52.86 \times FD^{2} ~} \hfill \\ {~\;\left( {1.86} \right)\quad \quad \left( {9.68} \right)\quad \quad \quad \left( {7.08} \right)\quad \quad \quad \quad \left( {11.38} \right)~~} \hfill \\ \end{array} \hfill \\ \quad {\text{AIC: 114}}{\text{.89}} \hfill \\ \end{gathered}$$

where values in parentheses indicate standard deviations. The same applies hereafter. Temperature (T) is included in the model not because it affects the occurrence of receptive females but because it affects the FD and/or FI of receptive females. The AIC value increased by 30, which is substantial, when temperature was excluded from the model.

Figure 2 shows the FD and FI of each flash from receptive females, mated females and males with the discriminant boundaries of receptive females from others for $p=0.5$.

We next considered a discriminant analysis for the experimental data. Let ${q}^{f}({\varvec{x}})$ denote the conditional probability that a flying male is attracted to a flash of ${\varvec{x}}=\left(\mathrm{FD},\mathrm{ FI},\mathrm{ T}\right)$ and lands, and ${\widehat{q}}^{f}({\varvec{x}})$ denote its estimate. Among several models we fit, the smallest AIC value is attained by the logistic regression model with FD, FI and T as explanatory variables, but the AIC is not much different from the model with FD and FI only.

**[Model for flying males with temperature (T)]**

$$\begin{gathered} {\text{log}}\frac{{\hat{q}^{f} }}{{1 - \hat{q}^{f} }} = \begin{array}{*{20}l} { - 0.74~~ - 2.42 \times FD - 16.82 \times FI + 0.31 \times T} \hfill \\ {~\;\left( {4.01} \right)\quad \quad \left( {0.83} \right)\quad \quad \quad \left( {4.88} \right)\quad \quad \quad \quad \left( {0.20} \right)~} \hfill \\ \end{array} \hfill \\ \quad {\text{AIC}}:66.96 \hfill \\ \end{gathered}$$

**[Model for flying males without temperature (T)]**

$$\begin{gathered} {\text{log}}\frac{{\hat{q}^{f} }}{{1 - \hat{q}^{f} }} = \begin{array}{*{20}l} { - 5.36~ - 1.72 \times FD - 13.69 \times FI} \hfill \\ {~\;\left( {1.49} \right)\quad \quad \left( {0.63} \right)~\quad \quad \left( {4.09} \right)~~} \hfill \\ \end{array} \hfill \\ \quad {\text{AIC}}:67.61 \hfill \\ \end{gathered}$$

For sedentary males, the model with the smallest AIC value includes all the quadratic terms of FI and FD but not temperature. Let ${q}^{s}({\varvec{x}})$ denote the conditional probability that a sedentary male is attracted to a flash of ${\varvec{x}}=\left(\mathrm{FD},\mathrm{ FI},\mathrm{ T}\right)$ and ${\widehat{q}}^{s}\left({\varvec{x}}\right)$ denote its estimate. The logistic regression model for ${q}^{s}({\varvec{x}})$ with the best AIC value is given as follows.

**[Model for sedentary males]**

$${\text{log}}\frac{{\hat{q}~^{s} }}{{1 - \hat{q}~^{s} }} = \begin{array}{*{20}l} { - 0.68~ + 7.84 \times FD~ + 48.17 \times FI - 5.35 \times FD^{2} - 166.70 \times FI^{2} - 65.67 \times FD \times FI} \hfill \\ {\;\left( {0.97} \right)\quad \quad \quad \left( {2.99} \right)\quad \quad \quad \left( {17.74} \right)\quad \quad \quad \left( {1.74} \right)\quad \quad \quad \quad \left( {72.34} \right)\quad \quad \quad \quad \left( {17.67} \right)~} \hfill \\ \end{array}$$

Figure 3 shows successes and failures of attraction of flying males on the left and sedentary males on the right with estimated discriminant boundaries.

Let us now estimate probabilities that a flying male is attracted and lands or a sedentary male is attracted to a flash when a flash is from a receptive female or when a flash is either from a sedentary male or mated female. The probability that a flying male is attracted and lands when a flash is from a receptive female is a conditional probability and is expressed as follows.

$$\begin{aligned} P\left(\left.\begin{array}{*{20}c} {\text{Flying male}} \\ {\text{is attracted}} \\ \end{array} \right|\begin{array}{*{20}c} {\text{Receptive }} \\ {{\text{female}}} \\ \end{array} \right) & = \frac{{P\left( {\begin{array}{*{20}c} {\text{Flying male}} \\ {\text{is attracted}} \\ \end{array} {\text{ and }}\begin{array}{*{20}c} {\text{Receptive }} \\ {{\text{female}}} \\ \end{array} } \right) }}{{P\left( {\begin{array}{*{20}c} {{\text{Receptive}}} \\ {{\text{female}}} \\ \end{array} } \right)}}, \\ P\left( {\begin{array}{*{20}c} {{\text{Receptive}}} \\ {{\text{female}}} \\ \end{array} } \right) & = \mathop \int_{\Omega } P\left(\left. \begin{array}{*{20}c} {{\text{Receptive}}} \\ {{\text{female}}} \\ \end{array} \right|{\varvec{x}} \right)f\left( {\varvec{x}} \right)d{\varvec{x}} = \mathop \int_{\Omega }p\left( {\varvec{x}} \right) f\left( {\varvec{x}} \right)d{\varvec{x}} \hspace{5mm}{\text{and}} \\ P\left( {\begin{array}{*{20}c} {\text{Flying male}} \\ {\text{is attracted}} \\ \end{array} {\text{ and }}\begin{array}{*{20}c} {\text{Receptive }} \\ {{\text{female}}} \\ \end{array} } \right) & = \mathop \int_{\Omega } P\left(\left. \begin{array}{*{20}c} {{\text{Receptive}}} \\ {{\text{female}}} \\ \end{array} \right|\varvec{x} \right)P\left(\left. \begin{array}{*{20}c} {\text{Flying male}} \\ {\text{is attracted}} \\ \end{array} \right|{\varvec{x}} \right)f\left( {\varvec{x}} \right)d{\varvec{x}} \\ & = \mathop \int_{\Omega } p\left( \varvec{x} \right)q^{f} \left( {\varvec{x}} \right)f\left( {\varvec{x}} \right)d{\varvec{x}}\mathbf{.} \\ \end{aligned}$$

Integrals are taken over the domain $\Omega$ of ${\varvec{x}}=(FD, FI, T)$ of all females and males, and $f({\varvec{x}})$ is the joint density function of ${\varvec{x}}.$ Because $f({\varvec{x}})$ is unknown, we use the empirical distribution of the observational data, and conditional probabilities given ${\varvec{x}}$ are replaced with their estimates by logistic regression models. Let ${{\varvec{x}}}_{i}=\left(F{D}_{i}, F{I}_{i}, {T}_{i}\right), i=\mathrm{1,2},\dots N$ denote the $i$ th observation in the observational data. The estimates of probabilities are given as follows:

$$\begin{aligned} \hat{P}\left( {\begin{array}{*{20}c} {{\text{Receptive}}} \\ {{\text{female}}} \\ \end{array} }\right) & = \frac{1}{N}\mathop \sum \limits_{i = 1}^{n} \hat{p}\left( {{\varvec{x}}_{i} } \right) \hspace{15mm} {\text{and}} \\ \hat{P}\left( {\begin{array}{*{20}c} {\text{Flying male}} \\ {\text{is attracted}} \\ \end{array} {\text{ and }}\begin{array}{*{20}c} {\text{Receptive }} \\ {{\text{female}}} \\ \end{array} } \right) & = \frac{1}{N}\mathop \sum \limits_{i = 1}^{n} \hat{p}\left( {{\varvec{x}}_{i} } \right) \hat{q}^{f} \left( {{\varvec{x}}_{i} } \right). \\ \end{aligned}$$

Thus,

$$\hat{P}\left( \left. \begin{array}{*{20}c} {\text{Flying male}} \\ {\text{is attracted}} \\ \end{array} \right| \begin{array}{*{20}c} {\text{Receptive }} \\ {\text{female}} \\ \end{array} \right) = \frac{{\mathop \sum \nolimits_{i = 1}^{n} \hat{p}\left( {{\varvec{x}}_{i} } \right) \hat{q}^{f} \left( {{\varvec{x}}_{i} } \right)}}{{\mathop \sum \nolimits_{i = 1}^{n}\hat{p}\left(\varvec{x}_i \right)}}.$$

Similarly, we have

$$\begin{aligned} \hat{P}\left( \left.\begin{array}{*{20}c} {\text{Flying male}} \\ {\text{is attracted}} \\ \end{array}\right| {\text{Others}} \right) & = \frac{{\mathop \sum \nolimits_{i = 1}^{n} (1 - \hat{p}\left( {{\varvec{x}}_{i} } \right)) \hat{q}^{f} \left( {{\varvec{x}}_{i} } \right)}}{{\mathop \sum \nolimits_{i = 1}^{n} (1 - \hat{p}\left( {{\varvec{x}}_{i} } \right))}} \\ \hat{P}\left( \left. \begin{array}{*{20}c} {\text{Sedentary male}} \\ {\text{is attracted}} \\ \end{array} \right| \begin{array}{*{20}c} {\text{Receptive }} \\ {\text{female}} \\ \end{array} \right)& = \frac{{\mathop \sum \nolimits_{i = 1}^{n} \hat{p}\left( {{\varvec{x}}_{i} } \right) \hat{q}^{s} \left( {{\varvec{x}}_{i} } \right)}}{{\mathop \sum \nolimits_{i = 1}^{n} \hat{p}\left( \varvec{x}_{i} \right)}}\hspace{15mm} {\text{ and}} \\\hat{P}\left(\left. \begin{array}{*{20}c} {\text{Sedentary male}} \\ {\text{is attracted}} \\ \end{array}\right| {\text{Others}} \right) & = \frac{{\mathop \sum \nolimits_{i = 1}^{n} \left( {1 - \hat{p}\left( \varvec{x}_{i} \right)} \right) \hat{q}^{s} \left( {\varvec{x}_{i} } \right)}}{\mathop \sum \nolimits_{i = 1}^{n} \left( {1 - \hat{p}\left( \varvec{x}_{i} \right)} \right)} . \\ \end{aligned}$$

The estimated probabilities are shown in Table 1.

**Table 1:** Estimated probabilities of a flying male and a sedentary male being attracted to flashes from a receptive female and from others.

	Flying male (model with T)			Flying male (model without T)		
	Attracted	Not attracted	Total	Attracted	Not attracted	Total
Receptive female	0.995	0.005	1	0.988	0.012	1
Others	0.581	0.419	1	0.666	0.334	1

	Sedentary male (model with T)			Sedentary male (model without T)		
	Attracted	Not attracted	Total	Attracted	Not attracted	Total
Receptive female	0.816	0.184	1	0.818	0.182	1
Others	0.157	0.843	1	0.157	0.843	1

## Results

### Field observations of courtship behavior

**Figure 1:**
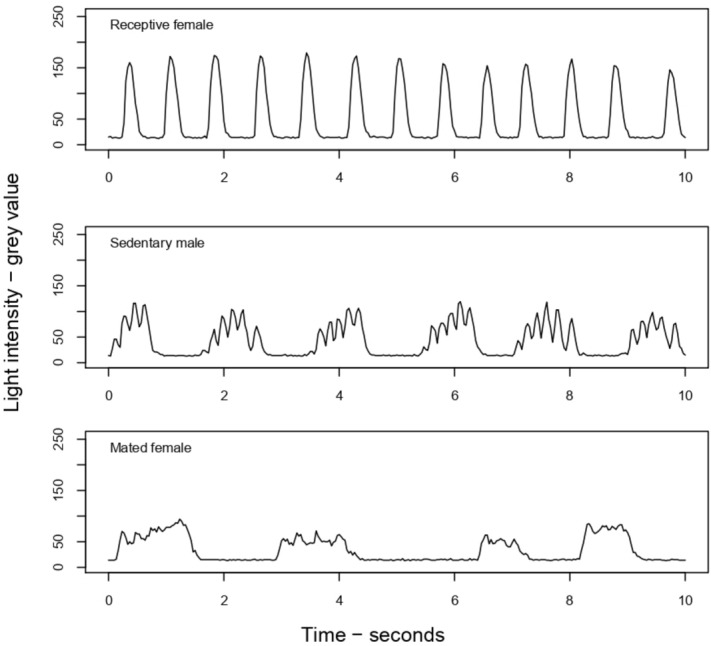
Examples of the waveforms from a receptive female (above), a sedentary male (middle), and a mated female (bottom) of adult *A. lateralis* recorded in the natural field.

Both sexes began to flash spontaneously 20–30 min after sunset. Males began to fly 40–50 min after sunset and usually stopped their flights 100 min after sunset. The ambient temperature during that period was 20.4–26.4 °C. Males perching on the vegetation in the daytime began to flash at dusk and approached and mounted sedentary females flashing spontaneously. Other males flying with flashes landed near sedentary females and immediately approached and mounted. The males were attracted not only to the receptive females but sometimes to other males and mated females. Flash exchange between males and females has been suggested^15,22^ and not suggested^14,23^, but we did not observe flash exchanges in our field. The flashes of the sedentary specimens, sedentary males, receptive females and mated females, were video-recorded (Fig. 1).

### Flash durations and flicker intensities of males and females

**Figure 2:**
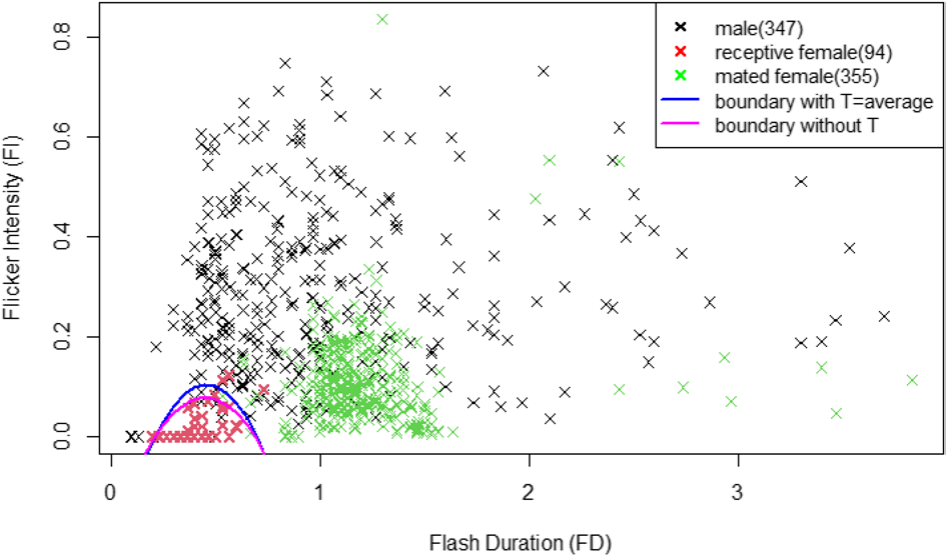
Flash duration (horizontal axis) and flicker intensity (vertical axis) of each flash from a receptive female (red), mated female (green) and male (black) with the discriminant boundaries of receptive females from others for *p* = 0.5 by the model with FD, FI, FD^2^ and T with T = the average temperature of 24.2 °C in blue and by the model with FD, FI and FD^2^ in magenta. The coordinate values of all points are shown in Supplementary Data S2.

The relationship between FD and FI for flashes of sedentary males, receptive females and mated females was analyzed to show the discriminant boundaries by a logistic regression model with and without temperature. The results showed that the flashes from receptive females could be discriminated from those from both males and mated females by FD and FI with little overlap (Fig. 2). In the receptive females, the FDs were generally shorter than those of both males and mated females, and the FIs were zero (76/94 flashes) or low (FI < 0.15) (Supplementary Data S2). In sedentary males, both FD and FI were higher with wide ranges than in the other groups. In the mated females, the FDs were longer and the FIs were higher than those of the receptive females (Fig. 2; see also Fig. 1). The discriminant boundaries with and without temperature were almost the same but slightly wider than those with temperature (Fig. 2).

### Male attraction experiments

**Figure 3:**
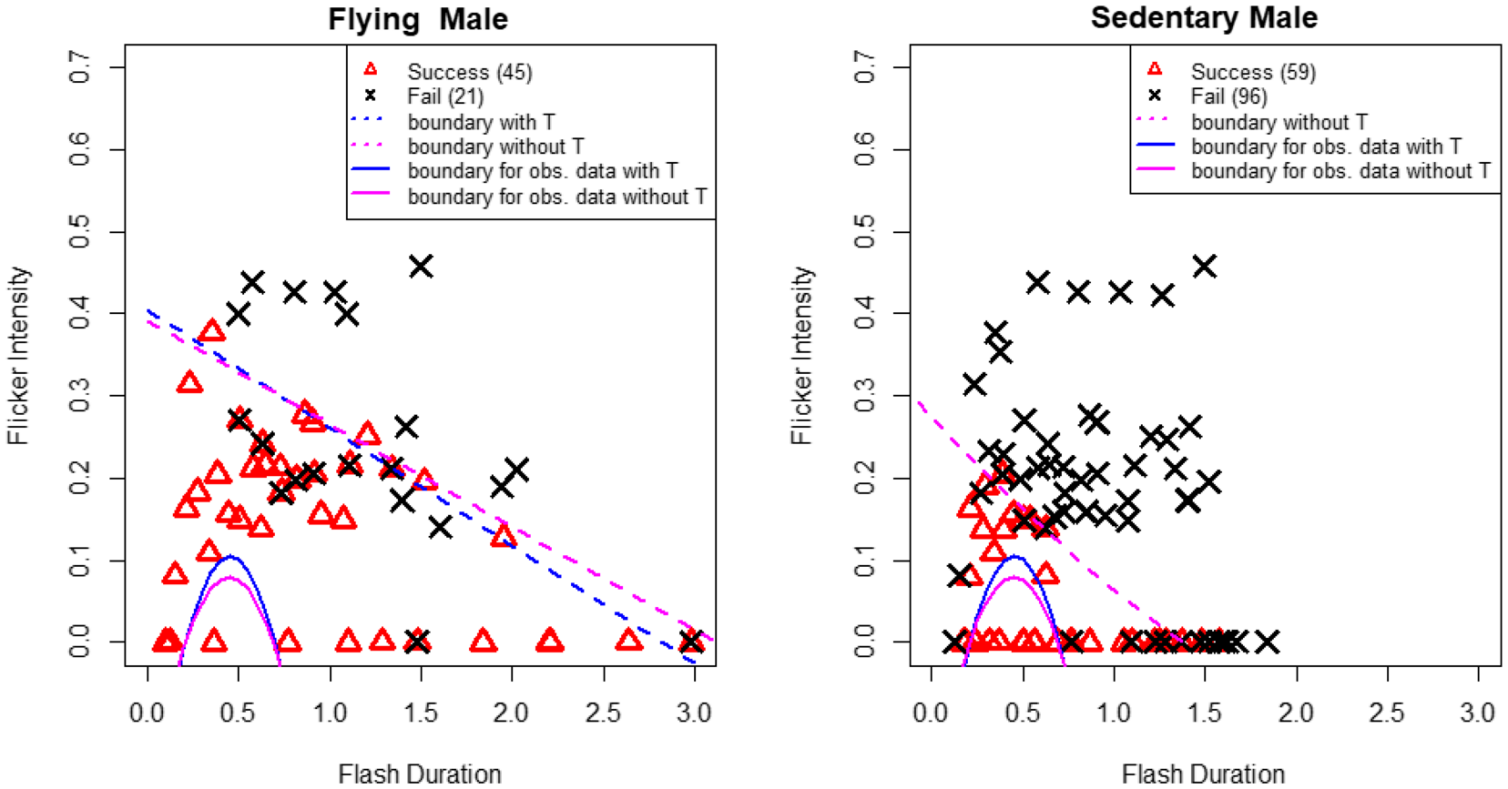
Successes (red dots) and failures (black cross) of attraction of flying males (left panel) and sedentary males (right panel) by e-firefly, with estimated discriminant boundaries for $p=0.5$. The blue dashed line is the estimated discriminant boundary by the model with temperature, and the magenta dashed line is that by the model without temperature. As references, the discriminant boundaries of receptive females from others for $p=0.5$ for the observational data are drawn in solid curves (see Fig. 1). The coordinate values of all points are shown in Supplementary Data S2.

The male attraction experiments using e-firefly showed that flashes with various ranges of FD and FI attracted flying and sedentary males (Fig. 3). The discriminant boundary of the flying males was much wider than those of flashes from the observed receptive females (Fig. 3, left panel). The discriminant boundary of the sedentary males was also wider but close to those of flashes from the observed receptive females (Fig. 3, right panel).

The statistical data analyses of the attraction experiments showed that almost 100% of the receptive females' signals attracted the flying males, but approximately half of the others’ (sedentary males and mated females) signals also attracted the flying males (Table 1). This is congruent with our frequent observations that the flying males landed near sedentary males. In contrast, approximately 80% of the receptive females’ signals attracted the sedentary males, but approximately 15% of the others’ signals also attracted the sedentary males (Table 1). Thus, our attraction experiments suggested that males can be attracted to other sedentary males and to mated females. In the field, indeed, males intending to mount and copulate with other males or mated females were sometimes observed. To the best of our knowledge, this is the second report to show a type of same-sex behavior in firefly species, but in the first case by Papi^17^, the flying male was attracted by sedentary male flashes. This suggests loose mate recognition in *A. lateralis*.

## Discussion

In the field, we observed three types of flash patterns of *A. lateralis* on the vegetation; the sedentary males emitted flashes with various durations and conspicuous flickering, as previously reported^15^. This time, we found that the flash pattern of females was dramatically changed after copulation; receptive females, which are scarce in the field compared to the other two types, emitted flashes with a short duration and very small or no flickering, as previously reported^15^, but after mating, they changed it to a longer duration and higher flickering flash pattern. When a flying male flew over the conspecific flashing on the vegetation, he landed near the light source, and this behavior seemed unselective to the flash patterns. In contrast, sedentary males approached exclusively in response to the short and nonflickering flashes of receptive females. Based on these preliminary field observations, we hypothesized the involvement of flickering as a parameter of mate recognition in *A. lateralis*.

To understand the flash signal system for mating in *A. lateralis*, we first video-recorded the flashes of sedentary males, receptive females and mated females in the field and analyzed each flash. The results showed that the flashes of the receptive females can be discriminated from those of both males and mated females by FD and FI with little overlap.

Then, we performed male attraction experiments using an artificial light source. An electronic LED device, e-firefly, was built to generate patterned flashes with different FDs and FIs. The color of the LED used for the e-firefly was ‘green’, which emits a broad spectrum ranging from approximately 530–600 nm (λmax = 568 nm) (Fig. S3. In *A. lateralis*, the adults in both sexes emits the same yellow–green color^15^, which has a broader spectrum ranging from approximately 450–700 nm (λmax = 554 nm) (Fig. S3), and their visual sensitivity was reported to be much broader with a peak at approximately 560 nm^15^. It is known that *A. lateralis* males are attracted to various colors from artificial light, such as cigarettes and car turn signals^14,22^. We therefore assessed that the difference in peak wavelength between the luminescence of the actual firefly and the LED of the e-firefly will not greatly affect the results of the attraction experiments. As a result, the flying males were attracted to the various flash waveforms of the e-firefly in the context of FD and FI, which included the range of receptive female signal parameters and extended much wider. In contrast, sedentary males were attracted to the narrower range of e-firefly flash patterns, which included the range of receptive female signal parameters but were not much extended; thus, flashes with longer FD and higher FI always failed to attract.

Taken together, our data demonstrated that, in addition to FD, which was also reported in other fireflies^10^, FI is a parameter of mate recognition in *A. lateralis*. This is the first report to demonstrate the involvement of flickering as a factor in the flash signals of fireflies. The wider discriminant boundary in flying males and the strict discrimination in sedentary males will be explained as a sort of stepwise recognition, or compensation, to scoop rivals and reduce mate-choice error. The primary function of bioluminescence in fireflies is considered to be an aposematic display; thus, flashing in the adult stage will always be advantageous. Now, what is the function of flickering in sedentary males? As *A. lateralis* is a female-advertising species, flickering in sedentary males is not used for mate calling but instead probably to restrain conspecific male competitors. The function of flickering in mated females will be to mimic sedentary males to avoid unwilling intervention by males.

Incidentally, can *A. lateralis* visually recognize the flickering of light? The flicker fusion frequency of fireflies was measured using the American nocturnal firefly, *Photinus pyralis*, and the highest value was approximately 40 Hz, which was almost the same as that in a nonluminous elateroid beetle (Cantharidae)^24^. This suggests that beetles generally can recognize the flickering of light at least lower than 40 Hz; thus, *A. lateralis* males can probably recognize conspecific flickering signals as well.

It was demonstrated that temporal factors in the flash signal, such as FD and response delay time, are the essential signal parameters in the bioluminescent sexual communication of various fireflies, including female-advertising species^10^. In contrast, nontemporal factors of firefly flashes have long been overlooked. In this study, we demonstrated for the first time that flicker intensity, a nontemporal factor, can also be a parameter of bioluminescent sexual communication in the Japanese aquatic firefly, *A. lateralis*. This sophisticated communication system, which involves both temporal and nontemporal factors, is comparable with sound communications such as the vibration call of frogs and the trill of song birds.

## Supplementary Information

Supplementary Information 1.

Supplementary Information 2.

Supplementary Information 3.

Supplementary Information 4.

## Data Availability

The dataset used and/or analyzed during the current study and the microcontroller program are available in Supplementary Data S1 and S2. Other data are available from the corresponding author upon reasonable request.
