# Supplementary material for: Flickering flash signals and mate recognition in the Asian firefly, Aquatica lateralis
Source: Sci Rep. 2023 Feb 10;13:2415. doi: 10.1038/s41598-023-29552-6 (PMC9918520; doi:10.1038/s41598-023-29552-6)
Supplement: Supplementary file 1 — Supplementary Information 1. [file 41598_2023_29552_MOESM1_ESM.docx]

**Supplementary Data S1.** An example of program for the microcontroller.

Program: FD=0.69, FI=0.14

;;;;;;;;;;;;;;;;;;;;;;;;;;;;;;;;;;;;;;;;;;;;;;;;;;;;;;;;;;;;;;;;;;;;;;;;;;;;;;;;;;;;;;;;;;;;;;;;;;;;;;;;;;;;;;;;;;;;;;;;;;;;;;;

list p=16f628a

#include <p16f628a.inc>

__CONFIG _CP_OFF & _BOREN_ON & _MCLRE_OFF & _WDT_OFF & _PWRTE_ON & _LVP_OFF & _INTOSC_OSC_NOCLKOUT ; Write this configuration word in one line.

w_temp EQU 0x70

status_temp EQU 0x71

CNT1 EQU 0x21

CNT2 EQU 0x22

CNT3 EQU 0x23

CNT4 EQU 0x24

CNT5 EQU 0x25

CNT6 EQU 0x26

CNT7 EQU 0x27

ORG 0x000

goto main

ORG 0x004

movwf w_temp

movf STATUS,w

movwf status_temp

movf status_temp,w

movwf STATUS

swapf w_temp,f

swapf w_temp,w

retfie

main

bcf STATUS,RP0

bcf STATUS,RP1

clrf INTCON

clrf PORTA

movlw 0x07

movwf CMCON

bsf STATUS,RP0

bsf PCON,OSCF

clrf TRISA

clrf TRISB

bcf STATUS,RP0

clrf PORTA

clrf PORTB

main_loop

btfss PORTA,0

goto response

RA0_HIGH

bcf PORTB,2

goto main_loop

response

movlw 0x2

movwf CNT1

loop1 bsf PORTB, 2

call delay_0.05

bcf PORTB,2

call delay_0.95

decfsz CNT1,f

goto loop1

movlw 0x2

movwf CNT1

loop2 bsf PORTB,2

call delay_0.1

bcf PORTB,2

call delay_0.9

decfsz CNT1,f

goto loop2

movlw 0x2

movwf CNT1

loop3 bsf PORTB,2

call delay_0.15

bcf PORTB,2

call delay_0.85

decfsz CNT1,f

goto loop3

movlw 0x2

movwf CNT1

loop4 bsf PORTB,2

call delay_0.2

bcf PORTB,2

call delay_0.8

decfsz CNT1,f

goto loop4

movlw 0x2

movwf CNT1

loop5 bsf PORTB,2

call delay_0.25

bcf PORTB,2

call delay_0.75

decfsz CNT1,f

goto loop5

movlw 0x2

movwf CNT1

loop6 bsf PORTB,2

call delay_0.3

bcf PORTB,2

call delay_0.7

decfsz CNT1,f

movlw 0x2

movwf CNT1

loop7 bsf PORTB,2

call delay_0.35

bcf PORTB,2

call delay_0.65

decfsz CNT1,f

goto loop7

movlw 0x2

movwf CNT1

loop8 bsf PORTB,2

call delay_0.4

bcf PORTB,2

call delay_0.6

decfsz CNT1,f

goto loop8

movlw 0x2

movwf CNT1

loop9 bsf PORTB,2

call delay_0.45

bcf PORTB,2

call delay_0.55

decfsz CNT1,f

goto loop9

movlw 0x2

movwf CNT1

loop10 bsf PORTB,2

call delay_0.5

bcf PORTB,2

call delay_0.5

decfsz CNT1,f

goto loop10

movlw 0x2

movwf CNT1

loop11 bsf PORTB,2

call delay_0.55

bcf PORTB,2

call delay_0.45

decfsz CNT1,f

goto loop11

movlw 0x2

movwf CNT1

loop12 bsf PORTB, 2

call delay_0.6

bcf PORTB,2

call delay_0.4

decfsz CNT1,f

goto l oop12

movlw 0x2

movwf CNT1

loop13 bsf PORTB, 2

call delay_0.65

bcf PORTB,2

call delay_0.35

decfsz CNT1,f

goto loop13

movlw 0x2

movwf CNT1

loop14 bsf PORTB,2

call delay_0.7

bcf PORTB,2

call delay_0.3

decfsz CNT1,f

goto loop14

movlw 0x2

movwf CNT1

loop15 bsf PORTB,2

call delay_0.75

bcf PORTB,2

call delay_0.25

decfsz CNT1,f

goto loop15

movlw 0x2

movwf CNT1

loop16 bsf PORTB,2

call delay_0.8

bcf PORTB,2

call delay_0.2

decfsz CNT1,f

goto loop16

movlw 0x2

movwf CNT1

loop17 bsf PORTB,2

call delay_0.85

bcf PORTB,2

call delay_0.15

decfsz CNT1,f

goto loop17

movlw 0x2

movwf CNT1

loop18 bsf PORTB,2

call delay_0.9

bcf PORTB,2

call delay_0.1

decfsz CNT1,f

goto loop18

movlw 0x2

movwf CNT1

loop19 bsf PORTB,2

call delay_0.95

bcf PORTB,2

call delay_0.05

decfsz CNT1,f

goto loop19

movlw 0x2

movwf CNT1

loop20 bsf PORTB,2

call delay_1

bcf PORTB,2

decfsz CNT1,f

goto loop20

movlw 0x2

movwf CNT1

loop21 bsf PORTB,2

call delay_0.95

bcf PORTB,2

call delay_0.05

decfsz CNT1,f

goto loop21

movlw 0x2

movwf CNT1

loop22 bsf PORTB,2

call delay_0.9

bcf PORTB,2

call delay_0.1

decfsz CNT1,f

goto loop22

movlw 0x2

movwf CNT1

loop23 bsf PORTB,2

call delay_0.85

bcf PORTB,2

call delay_0.15

decfsz CNT1, f

goto loop23

movlw 0x2

movwf CNT1

loop24 bsf PORTB,2

call delay_0.8

bcf PORTB,2

call delay_0.2

decfsz CNT1, f

goto l oop24

movlw 0x2

movwf CNT1

loop25 bsf PORTB,2

call delay_0.75

bcf PORTB,2

call delay_0.25

decfsz CNT1, f

goto loop25

movlw 0x2

movwf CNT1

loop26 bsf PORTB,2

call delay_0.7

bcf PORTB,2

call delay_0.3

decfsz CNT1,f

goto loop26

movlw 0x2

movwf CNT1

loop27 bsf PORTB,2

call delay_0.65

bcf PORTB,2

call delay_0.35

decfsz CNT1, f

goto loop27

movlw 0x2

movwf CNT1

loop28 bsf PORTB,2

call delay_0.6

bcf PORTB,2

call delay_0.4

decfsz CNT1,f

goto loop28

movlw 0x2

movwf CNT1

loop29 bsf PORTB,2

call delay_0.55

bcf PORTB,2

call delay_0.45

decfsz CNT1,f

goto loop29

movlw 0x2

movwf CNT1

loop30 bsf PORTB,2

call delay_0.5

bcf PORTB,2

call delay_0.5

decfsz CNT1,f

goto loop30

movlw 0x2

movwf CNT1

loop31 bsf PORTB, 2

call delay_0.45

bcf PORTB,2

call delay_0.55

decfsz CNT1,f

goto loop31

movlw 0x2

movwf CNT1

loop32 bsf PORTB, 2

call delay_0.4

bcf PORTB,2

call delay_0.6

decfsz CNT1,f

goto loop32

movlw 0x2

movwf CNT1

loop33 bsf PORTB,2

call delay_0.35

bcf PORTB,2

call delay_0.65

decfsz CNT1,f

goto loop33

movlw 0x2

movwf CNT1

loop34 bsf PORTB,2

call delay_0.3

bcf PORTB,2

call delay_0.7

decfsz CNT1, f

goto loop34

movlw 0x2

movwf CNT1

loop35 bsf PORTB,2

call delay_0.25

bcf PORTB,2

call delay_0.75

decfsz CNT1,f

goto loop35

movlw 0x2

movwf CNT1

loop36 bsf PORTB,2

call delay_0.3

bcf PORTB,2

call delay_0.7

decfsz CNT1,f

goto loop36

movlw 0x2

movwf CNT1

loop37 bsf PORTB,2

call delay_0.35

bcf PORTB,2

call delay_0.65

decfsz CNT1,f

goto loop37

movlw 0x2

movwf CNT1

loop38 bsf PORTB, 2

call delay_0.4

bcf PORTB,2

call delay_0.6

decfsz CNT1,f

goto loop38

movlw 0x2

movwf CNT1

loop39 bsf PORTB,2

call delay_0.45

bcf PORTB,2

call delay_0.55

decfsz CNT1,f

goto loop39

movlw 0x2

movwf CNT1

loop40 bsf PORTB,2

call delay_0.5

bcf PORTB,2

call delay_0.5

decfsz CNT1,

goto loop40

movlw 0x2

movwf CNT1

loop41 bsf PORTB,2

call delay_0.55

bcf PORTB,2

call delay_0.45

decfsz CNT1,f

goto loop41

movlw 0x2

movwf CNT1

loop42 bsf PORTB, 2

call delay_0.6

bcf PORTB,2

call delay_0.4

decfsz CNT1,f

goto loop42

movlw 0x2

movwf CNT1

loop43 bsf PORTB,2

call delay_0.65

bcf PORTB,2

call delay_0.35

decfsz CNT1,f

goto loop43

movlw 0x2

movwf CNT1

loop44 bsf PORTB,2

call delay_0.7

bcf PORTB,2

call delay_0.3

decfsz CNT1, f

goto loop44

movlw 0x2

movwf CNT1

loop45 bsf PORTB, 2

call delay_0.75

bcf PORTB,2

call delay_0.25

decfsz CNT1, f

goto loop45

movlw 0x2

movwf CNT1

loop46 bsf PORTB, 2

call delay_0.8

bcf PORTB,2

call delay_0.2

decfsz CNT1,f

goto loop46

movlw 0x2

movwf CNT1

loop47 bsf PORTB, 2

call delay_0.85

bcf PORTB,2

call delay_0.15

decfsz CNT1, f

goto loop47

movlw 0x2

movwf CNT1

loop48 bsf PORTB, 2

call delay_0.9

bcf PORTB,2

call delay_0.1

decfsz CNT1,f

goto loop48

movlw 0x2

movwf CNT1

loop49 bsf PORTB, 2

call delay_0.95

bcf PORTB, 2

call delay_0.05

decfsz CNT1,f

goto loop49

movlw 0x2

movwf CNT1

loop50 bsf PORTB, 2

call delay_1

bcf PORTB,2

decfsz CNT1,f

goto loop50

movlw 0x2

movwf CNT1

loop51 bsf PORTB, 2

call delay_0.95

bcf PORTB,2

call delay_0.05

decfsz CNT1, f

goto loop51

movlw 0x2

movwf CNT1

loop52 bsf PORTB, 2

call delay_0.9

bcf PORTB,2

call delay_0.1

decfsz CNT1,f

goto loop52

movlw 0x2

movwf CNT1

loop53 bsf PORTB, 2

call delay_0.85

bcf PORTB,2

call delay_0.15

decfsz CNT1, f

goto loop53

movlw 0x2

movwf CNT1

loop54 bsf PORTB, 2

call delay_0.8

bcf PORTB,2

call delay_0.2

decfsz CNT1, f

goto loop54

movlw 0x2

movwf CNT1

loop55 bsf PORTB, 2

call delay_0.75

bcf PORTB,2

call delay_0.25

decfsz CNT1,f

goto loop55

movlw 0x2

movwf CNT1

loop56 bsf PORTB, 2

call delay_0.7

bcf PORTB,2

call delay_0.3

decfsz CNT1,f

goto loop56

movlw 0x2

movwf CNT1

loop57 bsf PORTB, 2

call delay_0.65

bcf PORTB,2

call delay_0.35

decfsz CNT1, f

goto loop57

movlw 0x2

movwf CNT1

loop58 bsf PORTB, 2

call delay_0.6

bcf PORTB,2

call delay_0.4

decfsz CNT1,f

goto loop58

movlw 0x2

movwf CNT1

loop59 bsf PORTB, 2

call delay_0.55

bcf PORTB,2

call delay_0.45

decfsz CNT1,f

goto loop59

movlw 0x2

movwf CNT1

loop60 bsf PORTB, 2

call delay_0.5

bcf PORTB,2

call delay_0.5

decfsz CNT1, f

goto loop60

movlw 0x2

movwf CNT1

loop61 bsf PORTB, 2

call delay_0.45

bcf PORTB,2

call delay_0.55

decfsz CNT1, f

goto loop61

movlw 0x2

movwf CNT1

loop62 bsf PORTB, 2

call delay_0.4

bcf PORTB,2

call delay_0.6

decfsz CNT1,f

goto loop62

movlw 0x2

movwf CNT1

loop63 bsf PORTB, 2

call delay_0.35

bcf PORTB,2

call delay_0.65

decfsz CNT1, f

goto loop63

movlw 0x2

movwf CNT1

loop64 bsf PORTB, 2

call delay_0.3

bcf PORTB,2

call delay_0.7

decfsz CNT1, f

goto loop64

movlw 0x2

movwf CNT1

loop65 bsf PORTB, 2

call delay_0.25

bcf PORTB,2

call delay_0.75

decfsz CNT1, f

goto loop65

movlw 0x2

movwf CNT1

loop66 bsf PORTB, 2

call delay_0.3

bcf PORTB, 2

call delay_0.7

decfsz CNT1, f

goto loop66

movlw 0x2

movwf CNT1

loop67 bsf PORTB, 2

call delay_0.35

bcf PORTB,2

call delay_0.65

decfsz CNT1, f

goto loop67

movlw 0x2

movwf CNT1

loop68 bsf PORTB, 2

call delay_0.4

bcf PORTB,2

call delay_0.6

decfsz CNT1,f

goto loop68

movlw 0x2

movwf CNT1

loop69 bsf PORTB, 2

call delay_0.45

bcf PORTB,2

call delay_0.55

decfsz CNT1,f

goto loop69

movlw 0x2

movwf CNT1

loop70 bsf PORTB, 2

call delay_0.5

bcf PORTB,2

call delay_0.5

decfsz CNT1, f

goto loop70

movlw 0x2

movwf CNT1

loop71 bsf PORTB, 2

call delay_0.55

bcf PORTB,2

call delay_0.45

decfsz CNT1, f

goto loop71

movlw 0x2

movwf CNT1

loop72 bsf PORTB, 2

call delay_0.6

bcf PORTB,2

call delay_0.4

decfsz CNT1,f

goto loop72

movlw 0x2

movwf CNT1

loop73 bsf PORTB, 2

call delay_0.65

bcf PORTB,2

call delay_0.35

decfsz CNT1, f

goto loop73

movlw 0x2

movwf CNT1

loop74 bsf PORTB, 2

call delay_0.7

bcf PORTB,2

call delay_0.3

decfsz CNT1, f

goto loop74

movlw 0x2

movwf CNT1

loop75 bsf PORTB, 2

call delay_0.75

bcf PORTB,2

call delay_0.25

decfsz CNT1,f

goto loop75

movlw 0x2

movwf CNT1

loop76 bsf PORTB, 2

call delay_0.8

bcf PORTB,2

call delay_0.2

decfsz CNT1,f

goto loop76

movlw 0x2

movwf CNT1

loop77 bsf PORTB, 2

call delay_0.85

bcf PORTB,2

call delay_0.15

decfsz CNT1, f

goto loop77

movlw 0x2

movwf CNT1

loop78 bsf PORTB, 2

call delay_0.9

bcf PORTB,2

call delay_0.1

decfsz CNT1,f

goto loop78

movlw 0x2

movwf CNT1

loop79 bsf PORTB, 2

call delay_0.95

bcf PORTB,2

call delay_0.05

decfsz CNT1,f

goto loop79

movlw 0x2

movwf CNT1

loop80 bsf PORTB, 2

call delay_1

bcf PORTB,2

decfsz CNT1,f

goto loop80

movlw 0x2

movwf CNT1

loop81 bsf PORTB, 2

call delay_0.95

bcf PORTB,2

call delay_0.05

decfsz CNT1, f

goto loop81

movlw 0x2

movwf CNT1

loop82 bsf PORTB, 2

call delay_0.9

bcf PORTB,2

call delay_0.1

decfsz CNT1,f

goto loop82

movlw 0x2

movwf CNT1

loop83 bsf PORTB, 2

call delay_0.85

bcf PORTB,2

call delay_0.15

decfsz CNT1, f

goto loop83

movlw 0x2

movwf CNT1

loop84 bsf PORTB, 2

call delay_0.8

bcf PORTB,2

call delay_0.2

decfsz CNT1, f

goto loop84

movlw 0x2

movwf CNT1

loop85 bsf PORTB, 2

call delay_0.75

bcf PORTB,2

call delay_0.25

decfsz CNT1,f

goto loop85

movlw 0x2

movwf CNT1

loop86 bsf PORTB, 2

call delay_0.7

bcf PORTB,2

call delay_0.3

decfsz CNT1,f

goto loop86

movlw 0x2

movwf CNT1

loop87 bsf PORTB, 2

call delay_0.65

bcf PORTB,2

call delay_0.35

decfsz CNT1, f

goto loop87

movlw 0x2

movwf CNT1

loop88 bsf PORTB, 2

call delay_0.6

bcf PORTB,2

call delay_0.4

decfsz CNT1,f

goto loop88

movlw 0x2

movwf CNT1

loop89 bsf PORTB, 2

call delay_0.55

bcf PORTB,2

call delay_0.45

decfsz CNT1,f

goto loop89

movlw 0x2

movwf CNT1

loop90 bsf PORTB, 2

call delay_0.5

bcf PORTB,2

call delay_0.5

decfsz CNT1,f

goto loop90

movlw 0x2

movwf CNT1

loop91 bsf PORTB, 2

call delay_0.45

bcf PORTB,2

call delay_0.55

decfsz CNT1,f

goto loop91

movlw 0x2

movwf CNT1

loop92 bsf PORTB, 2

call delay_0.4

bcf PORTB,2

call delay_0.6

decfsz CNT1,f

goto loop92

movlw 0x2

movwf CNT1

loop93 bsf PORTB, 2

call delay_0.35

bcf PORTB,2

call delay_0.65

decfsz CNT1,f

goto loop93

movlw 0x2

movwf CNT1

loop94 bsf PORTB, 2

call delay_0.3

bcf PORTB,2

call delay_0.7

decfsz CNT1,f

goto loop94

movlw 0x2

movwf CNT1

loop95 bsf PORTB, 2

call delay_0.25

bcf PORTB,2

call delay_0.75

decfsz CNT1,f

goto loop95

movlw 0x2

movwf CNT1

loop96 bsf PORTB, 2

call delay_0.2

bcf PORTB,2

call delay_0.8

decfsz CNT1,f

goto loop96

movlw 0x2

movwf CNT1

loop97 bsf PORTB,2

call delay_0.15

bcf PORTB,2

call delay_0.85

decfsz CNT1,f

goto loop97

movlw 0x2

movwf CNT1

loop98 bsf PORTB, 2

call delay_0.1

bcf PORTB,2

call delay_0.9

decfsz CNT1,f

goto loop98

movlw 0x2

movwf CNT1

loop99 bsf PORTB, 2

call delay_0.05

bcf PORTB,2

call delay_0.95

decfsz CNT1,f

goto loop99

call delay_1200

goto response

delay_0.05

movlw 0x8

movwf CNT2

s_loop

nop

nop

decfsz CNT2,f

goto s_loop

return

delay_0.1

call delay_0.05

call delay_0.05

return

delay_0.15

call delay_0.05

call delay_0.1

return

delay_0.2

call delay_0.1

call delay_0.1

return

delay_0.25

call delay_0.05

call delay_0.2

return

delay_0.3

call delay_0.1

call delay_0.2

return

delay_0.35

call delay_0.05

call delay_0.3

return

delay_0.4

call delay_0.2

call delay_0.2

return

delay_0.45

call delay_0.05

call delay_0.4

return

delay_0.5

call delay_0.1

call delay_0.4

return

delay_0.55

call delay_0.05

call delay_0.5

return

delay_0.6

call delay_0.3

call delay_0.3

return

delay_0.65

call delay_0.05

call delay_0.6

return

delay_0.7

call delay_0.3

call delay_0.4

return

delay_0.75

call delay_0.05

call delay_0.7

return

delay_0.8

call delay_0.4

call delay_0.4

return

delay_0.85

call delay_0.05

call delay_0.8

return

delay_0.9

call delay_0.4

call delay_0.5

return

delay_0.95

call delay_0.05

call delay_0.9

return

delay_1

call delay_0.05

call delay_0.95

return

delay_1200

movlw 0xc

movwf CNT4

loopt

call delay_100

decfsz CNT4,f

goto loopt

return

delay_100

movlw 0x53

movwf CNT5

loops_1

movlw 0xf0

movwf CNT6

loops_2

nop

nop

decfsz CNT6, f

goto loops_2

decfsz CNT5,f

goto loops_1

return

end

;;;;;;;;;;;;;;;;;;;;;;;;;;;;;;;;;;;;;;;;;;;;;;;;;;;;;;;;;;;;;;;;;;;;;;;;;;;;;;;;;;;;;;;;;;;;;;;;;;;;;;;;;;;;;;;;;;;;;;;;;;;;;;;
