# Supplementary material for: Flickering flash signals and mate recognition in the Asian firefly, Aquatica lateralis
Source: Sci Rep. 2023 Feb 10;13:2415. doi: 10.1038/s41598-023-29552-6 (PMC9918520; doi:10.1038/s41598-023-29552-6)
Supplement: Supplementary file 3 — Supplementary Information 3. [file 41598_2023_29552_MOESM3_ESM.docx]

**Supplementary Text**

**Flickering flash signals and mate recognition in the Asian firefly, *Aquatica lateralis***

Hideo Takatsu^1,2^, Mihoko Minami^3^, Yuichi Oba^2*^

^1^ 5-58 Takayokosuka-machi, Tokai, Aichi, 477-0037, Japan

^2^ Department Environmental Biology, Chubu University, 1200 Matsumoto-cho, Kasugai, Aichi 487-8501, Japan

^3^ Department of Mathematics, Keio University, 3-14-1 Hiyoshi, Kohoku-ku, Yokohama, Kanagawa 223-8522, Japan

*Corresponding author: Yuichi Oba

Email address: [yoba@isc.chubu.ac.jp](mailto:yoba@isc.chubu.ac.jp)

All data plotted for Figures 2 and 3 are shown in Supplementary Data S2. Sheets ‘OBmale’, ‘OBmated’, ‘OBreceptive’ show the parameter values observed for the flashes of sedentary males, mated females and receptive females, respectively. Sheets ‘ele-success (sedentary)’, ‘ele-failure (sedentary)’, ‘ele-success (flying)’, ‘ele-failure (flying)’ show the parameter values generated by e-firefly for attraction success, failure of sedentary male’s attraction, failure of flying males, respectively. T, temperature; FD, flash duration; FI, flicker intensity.
