# Supplementary material for: Flickering flash signals and mate recognition in the Asian firefly, Aquatica lateralis
Source: Sci Rep. 2023 Feb 10;13:2415. doi: 10.1038/s41598-023-29552-6 (PMC9918520; doi:10.1038/s41598-023-29552-6)
Supplement: Supplementary file 4 — Supplementary Information 4. [file 41598_2023_29552_MOESM4_ESM.pdf]

Supplementary Figures

Flickering flash signals and mate recognition in the Asian firefly, *Aquatica lateralis*

Hideo Takatsu[1,2], Mihoko Minami[3], Yuichi Oba[2*]

[1] 5-58 Takayokosuka-machi, Tokai, Aichi, 477-0037, Japan
[2] Department Environmental Biology, Chubu University, 1200 Matsumoto-cho, Kasugai, Aichi 487-8501, Japan
[3] Department of Mathematics, Keio University, 3-14-1 Hiyoshi, Kohoku-ku, Yokohama, Kanagawa 223-8522, Japan

*Corresponding author: Yuichi Oba
Email address: yoba@isc.chubu.ac.jp

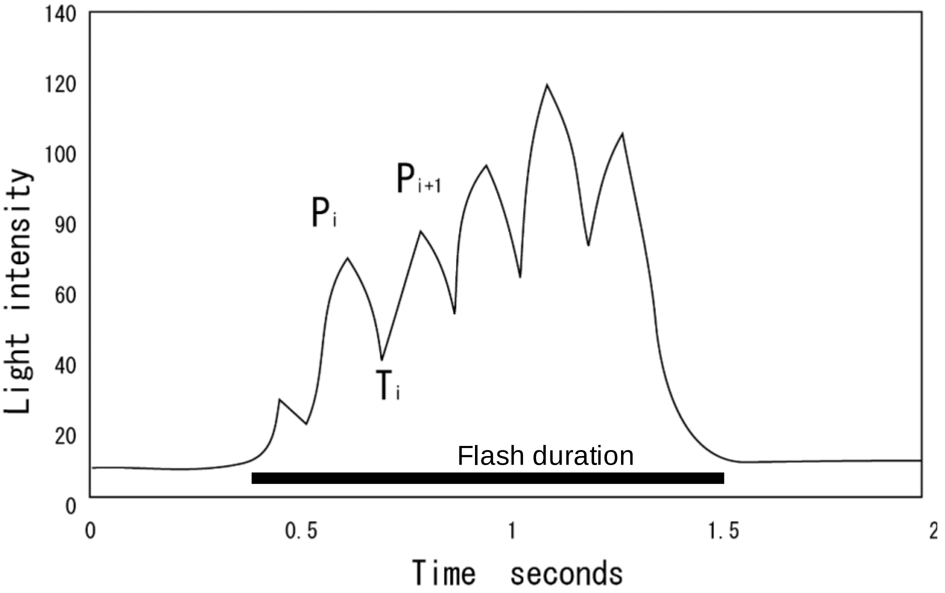

Supplementary Figure S1. Measurement of and flicker intensity. The P, T, and i indicate the light intensity of peak and trough in the waveform of a flash, and the ordinal number of troughs in the flash, respectively.

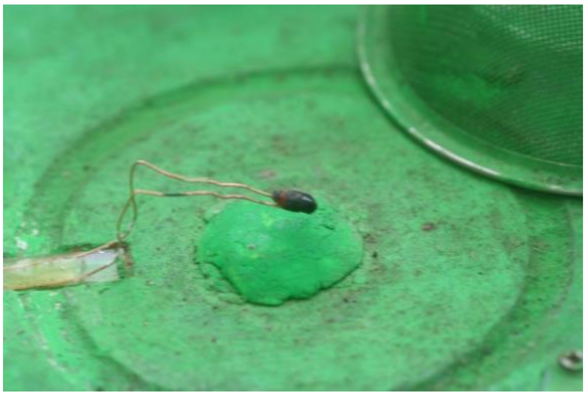

Supplementary Figure S2. LED chip (center) and painted net (right) of e-firefly. The diameter of the net is 75 mm.

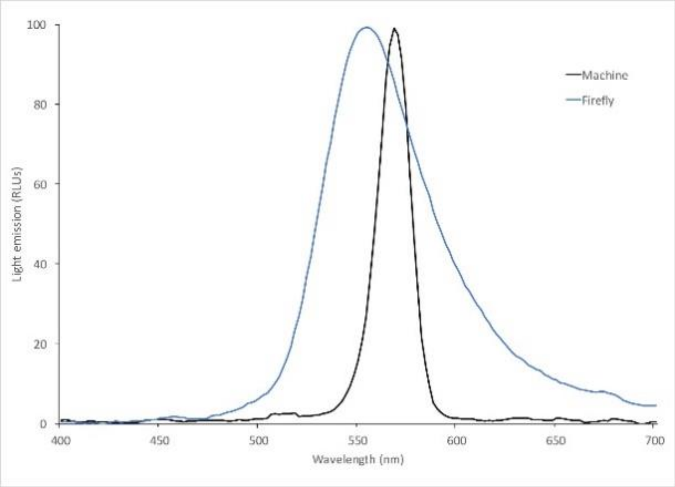

Supplementary Figure S3. Light emission spectra of e-firefly (black line) and *Aquatica lateralis* specimen (dotted line).

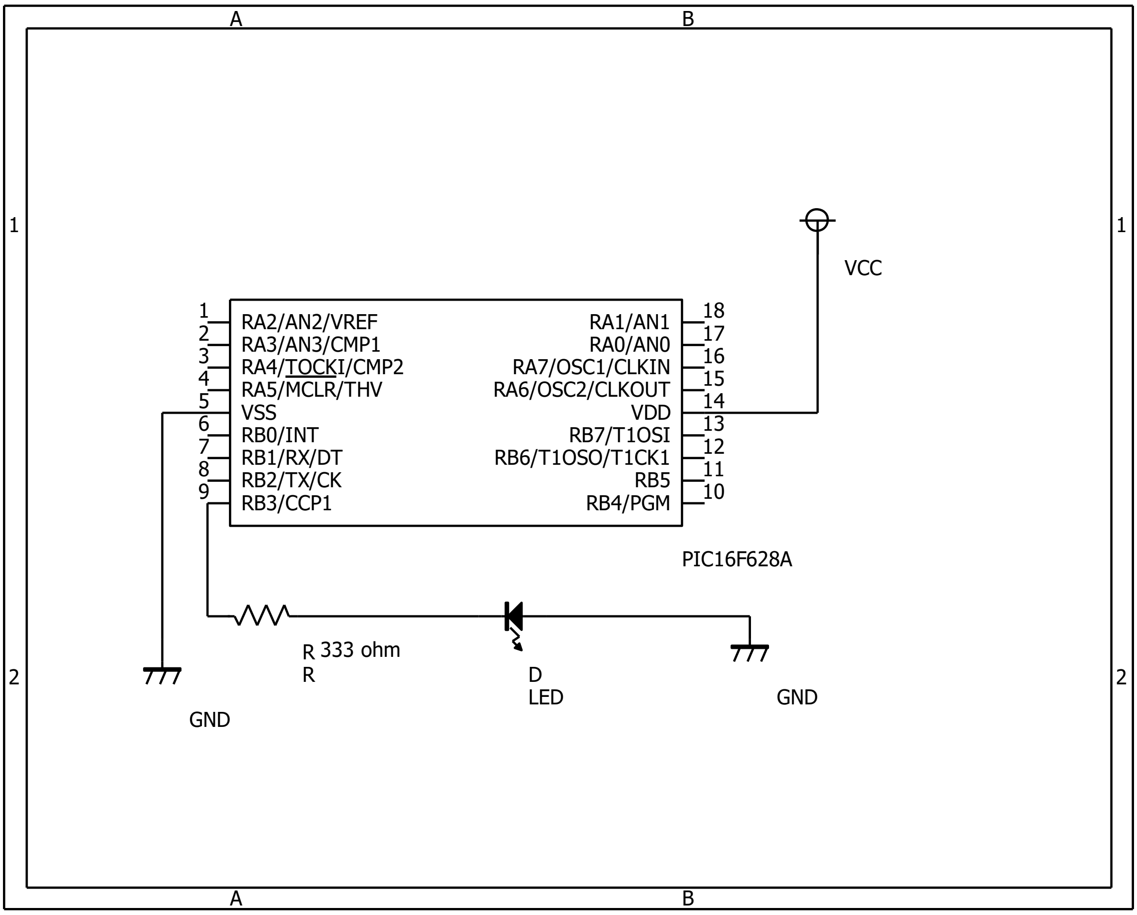

Supplementary Figure S4. Electronic circuit for the controlling of the e-firefly's LED. The microcontroller in the circuit is PIC16F628A (Microchip tec.).

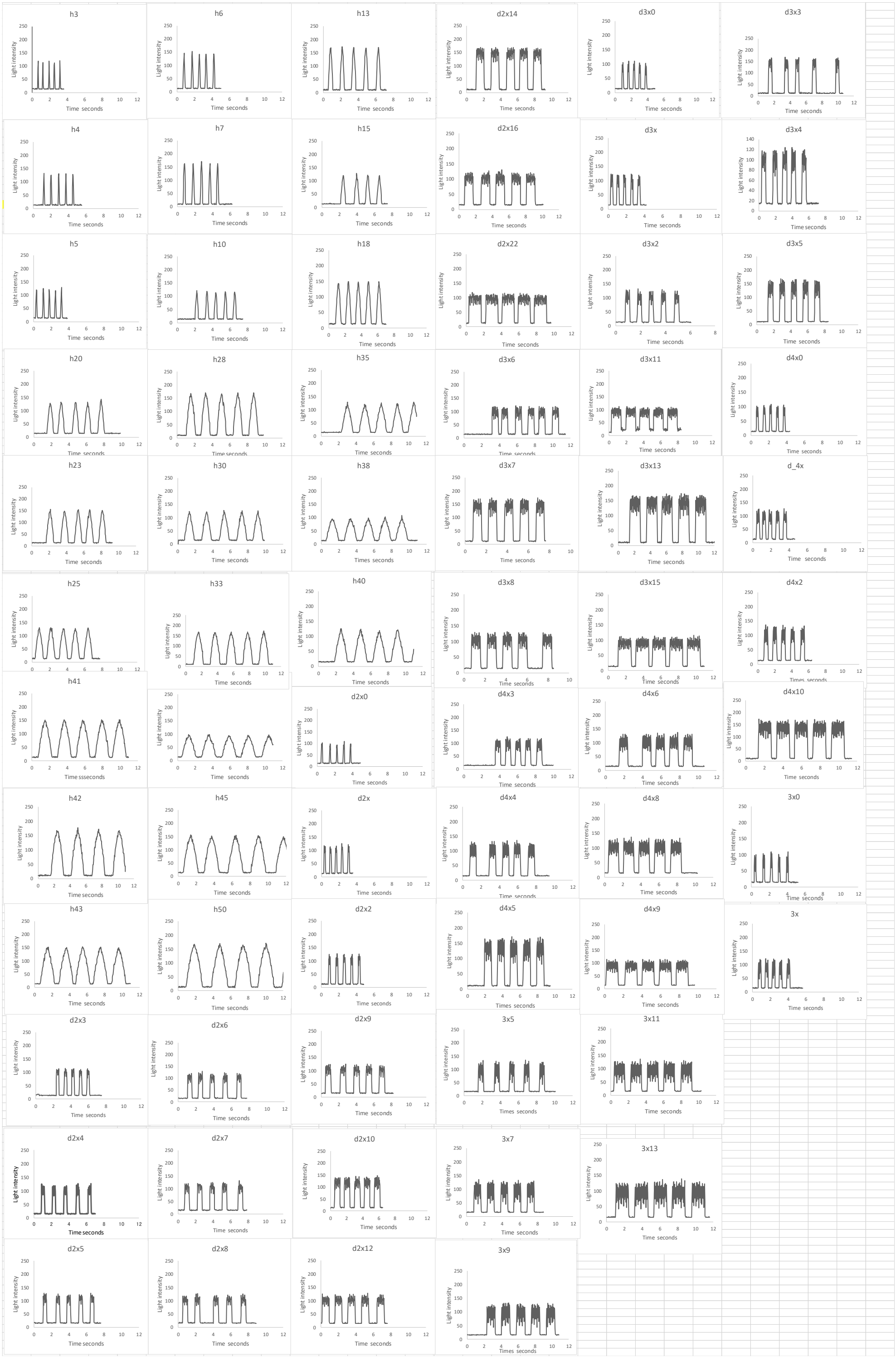

Supplementary Figure S5. Examples of the flash pattern generated by e-firefly.
